# Evaluation of urinary catheters for effective manual bladder washout

**DOI:** 10.1038/s41598-022-18778-5

**Published:** 2022-08-23

**Authors:** Kohei Kobatake, Shogo Inoue, Kenshiro Takemoto, Takahumi Fukushima, Yohei Sekino, Kenichiro Ikeda, Keisuke Goto, Tetsutaro Hayashi, Jun Teishima, Nobuyuki Hinata

**Affiliations:** 1 Department of Urology, Graduate School of Biomedical and Health Sciences, Hiroshima University, 1-2-3 Kasumi, Minami-ku, Hiroshima 734-8553 Japan; 2 Department of Urology, Mazda Motor Corporation, Mazda Hospital, 2-15 Aosaki-minami, Fuchu-cho, Aki-gun, Hiroshima 735-8585 Japan; 3 Department of Urology, Graduate School of Biomedical and Health Sciences, Hiroshima University, 1-2-3 Kasumi, Minami-ku, Hiroshima 734-8551 Japan

**Keywords:** Bladder, Biomedical engineering, Medical research, Urology

## Abstract

Our purpose was to evaluate the efficiency of manual bladder washout (MBW) for bladder retention by blood clot formation using urinary catheters. Three types of 22 Fr urinary catheters, a rounded tip Foley catheter (FC) with the standard two eyes, an open-ended Nelaton catheter (NC) with a side hole, and an open-ended Foley catheter (OEFC) with a side hole closer to the tip than NC, were evaluated. An automatic irrigation device that could perform a predetermined procedure mimicking MBW under constant velocity was fabricated. The procedure using catheters and the device was performed in a pseudo blood clot or in water. The total area of the holes was the largest in NC followed by FC and OEFC. The predetermined operations using our device revealed that NC needed less force and could effectively remove pseudo clots from the early stage of the operations. Fluid visualization experiments suggested that a closer distance between the tip and the side hole could improve the efficiency of clot removal. In conclusion, the larger the area of the hole in urinary catheter, the less force is required for MBW. Furthermore, the most efficient catheter with two holes for MBW needs to be at least open-ended with a side hole closer to the tip.

## Introduction

Gross hematuria is claimed to involve 4% of all urological visits^1^. Occasionally, it causes urinary retention by blood clot formation, which is a common emergency in urological practice. Patients may develop severe pain if the clots are not evacuated in a timely manner^2^. Blood clot retention can develop various symptoms, including tachycardia, hypertension, bladder rupture, and perforation from an acutely overdistended bladder^3^.

The first report of manual bladder washout (MBW) involved the use of 24 Fr Brown-Buerger cystoscopic sheath for bladder clot retention^4^ in 1929. Currently, MBW through a Foley catheter and syringe is the most common and simple method of removing such blood clots^2^. The procedure involves insertion of a urinary catheter into the bladder via the urethra, followed by bladder irrigation using a 60 ml catheter tip syringe filled with a saline solution until the clot is removed^5^. A recently developed standard protocol for MBW (CATCH-22) recommends the use of a minimum of 22 Fr catheter^6^. Several reports have shown the usefulness of catheters with additional side holes or catheters for non-urological settings such as thoracic and rectal tube^5,7,8^. In addition, the flow characteristics of various urinary catheters have been evaluated. However, due to the nature of emergency diseases, an accurate comparison of the efficiency of MBW using each catheter is quite difficult in patients with urinary retention due to blood clot formation. There was no report which has quantitatively evaluated the efficiency of urologic catheters in removing blood clots. This study aimed to compare and evaluate the efficiency of MBW using commercially available urinary catheters in vitro. Our results will aid in the development of novel catheters specifically for urinary retention by blood clot formation.

## Results

### Efficiency of MBW by urologists

**Figure 1:**
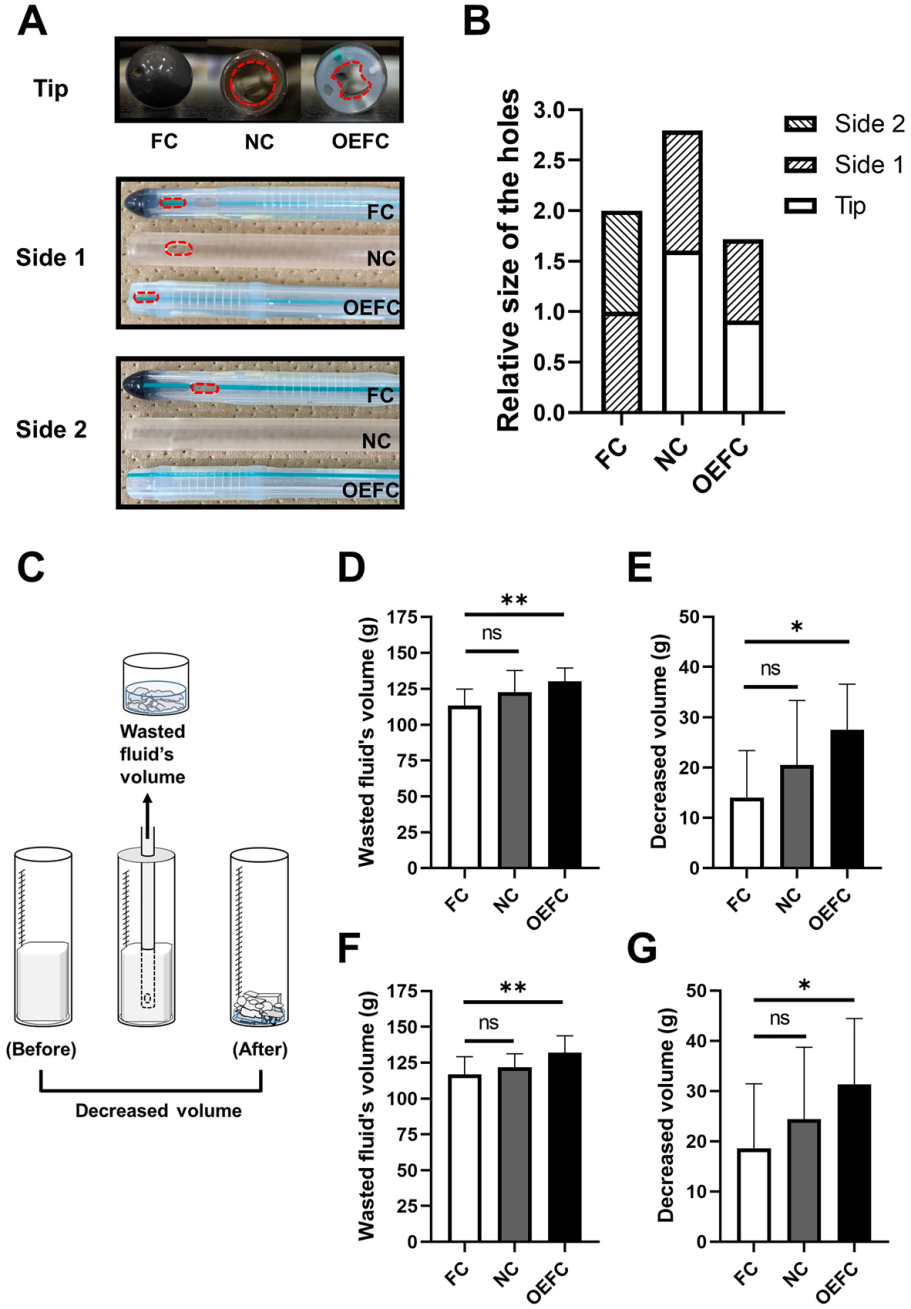
The efficiency of MBW. (**A**) Photographs viewed from the tip or each side of the catheters used in this study. Red dotted line shows the outline of each hole. (**B**) Total relative area of the holes in each catheter. The area of one of the holes in FC was set to 1. (**C**) The schematic image of how to evaluate MBW. (**D**,**E**) Wasted fluid’s volume and decreased clot’s volume of soft tofu. (**F**,**G**) Wasted fluid’s volume and decreased clot’s volume of hard tofu. The decreased volume of PBC after the procedure mimicking MBW was done by 11 urologists.

Three types of 22 Fr urinary catheters, a rounded tip Foley catheter (FC) with the standard two eyes, an open-ended Nelaton catheter (NC) with a side hole, and an open-ended Foley catheter (OEFC) with a side hole closer to the tip than NC, were evaluated. Photographs viewed from the tip or each side of the catheter were shown (Fig. 1A). The red dotted line shows the outline of each hole. The side holes were located at 9 and 17 mm respectively from the tip in FC, 8 mm in NC, and 2 mm in OEFC. The cross-section of FC was the same as that of OEFC, and each major axis of the lumen was 4 mm, while NC had 5 mm lumen diameter. Total relative area of the holes in each catheter is shown (Fig. 1B). Area of one of the holes in FC was set to 1. On comparing the area, NC had the largest holes followed by FC and OEFC.

The MBW procedure described in the “Materials and methods” section was performed using 2 types of pseudo blood clots (PBC), soft and hard tofu, by 11 urologists affiliated with Hiroshima University Hospital. The schematic image of how to evaluate MBW was shown in Fig. 1C. The wasted fluid’s volumes (Fig. 1D, *p* = 0.0044) and decreased clot’s volumes (Fig. 1E, *p* = 0.0129) were significantly greater in OEFC than FC when soft tofu was used. Equivalent results were observed when hard tofu was used (Fig. 1F, *p* = 0.0075 and Fig. 1G, *p* = 0.0324).

### Force required for irrigation

**Figure 2:**
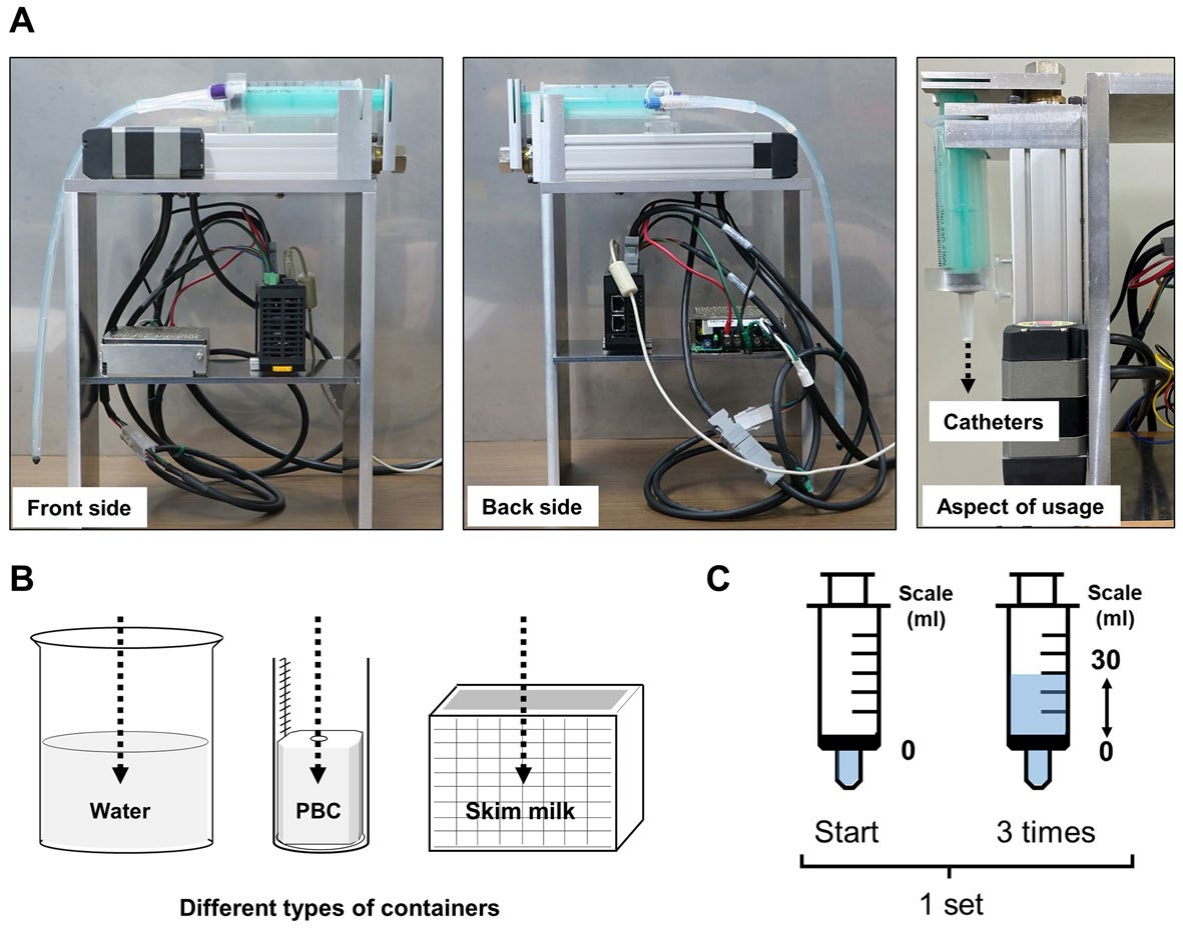
An automatic irrigation device. An automatic irrigation device for a 60 ml catheter tip syringe was used to repeat a predetermined movement at a constant velocity. (**A**) Detailed photographs of the device. (**B**) The tip of each catheter, attached to a syringe, was inserted into different types of containers appropriate for the experiment. (**C**) The syringe was set to start operation from the 0 scale, pulled to 30 scale, and pushed to 0 scale, repeating the operation three times. This series of operations was considered in a single set.

For a more precise evaluation of the efficiency of MBW using each catheter, we fabricated an automatic irrigation device for a 60 ml catheter tip syringe. Detailed image of the device and schematic image of our methodology using it were shown in Fig. 2A–C. Video of the actual operation can be seen in Supplementary information. At a velocity of 20 mm per second (mm/s), for example, the syringe moved about 13.5 ml syringe scale per second (ml/s), and the pulling time was completely consistent with the pushing time: between the ranges of 2159 and 2190 ms (ms).

**Figure 3:**
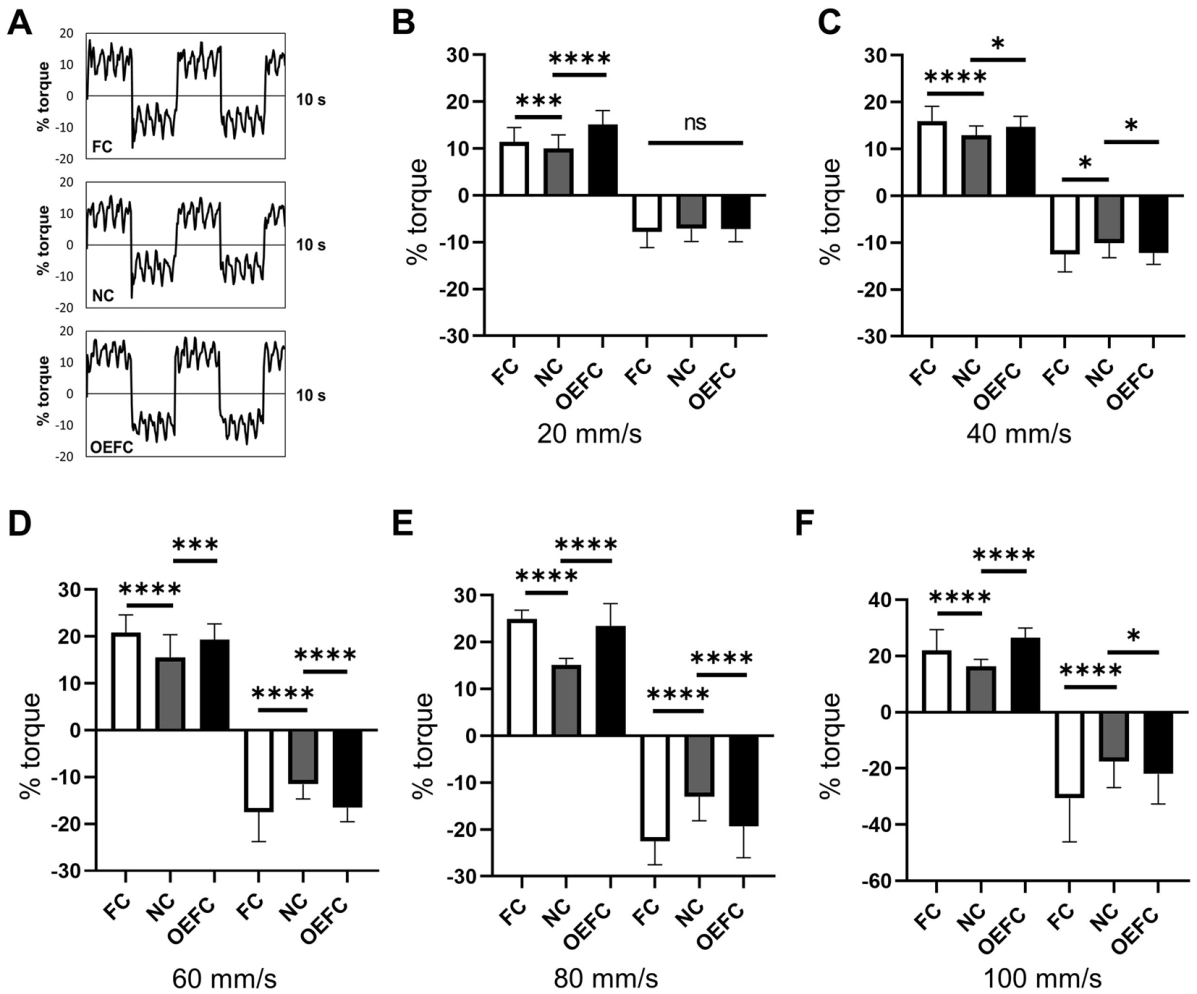
Force required for irrigation. (**A**) Images of the output waveform of the required force (% torque) when the operation was performed at 20 mm/s in water. The force required in the pulling direction was indicated as plus and the force required in the pushing direction was indicated as minus. (**B**–**F**) Multiple comparisons of average values of the force required for pulling and pushing at a velocity of 20, 40, 60, 80, and 100 mm/s.

One set of predetermined actions for the device was performed in water with each catheter attached to the device. The force output waveform when the operation was performed at 20 ml/s is shown (Fig. 3A). The force required in the pulling and pushing directions was indicated as plus and minus, respectively. For each catheter, we compared the magnitude of the force required for pulling and pushing at velocities of 20, 40, 60, 80, and 100 mm/s (Fig. 3B–F). Except at velocity of 20 mm/s for pushing, at all velocities, the average force required for both pulling and pushing was significantly different in the 3 catheters. Multiple comparison after 1-way ANOVA showed that the force was the smallest for NC (Fig. 3B–F).

### Deviation of catheter tip

**Figure 4:**
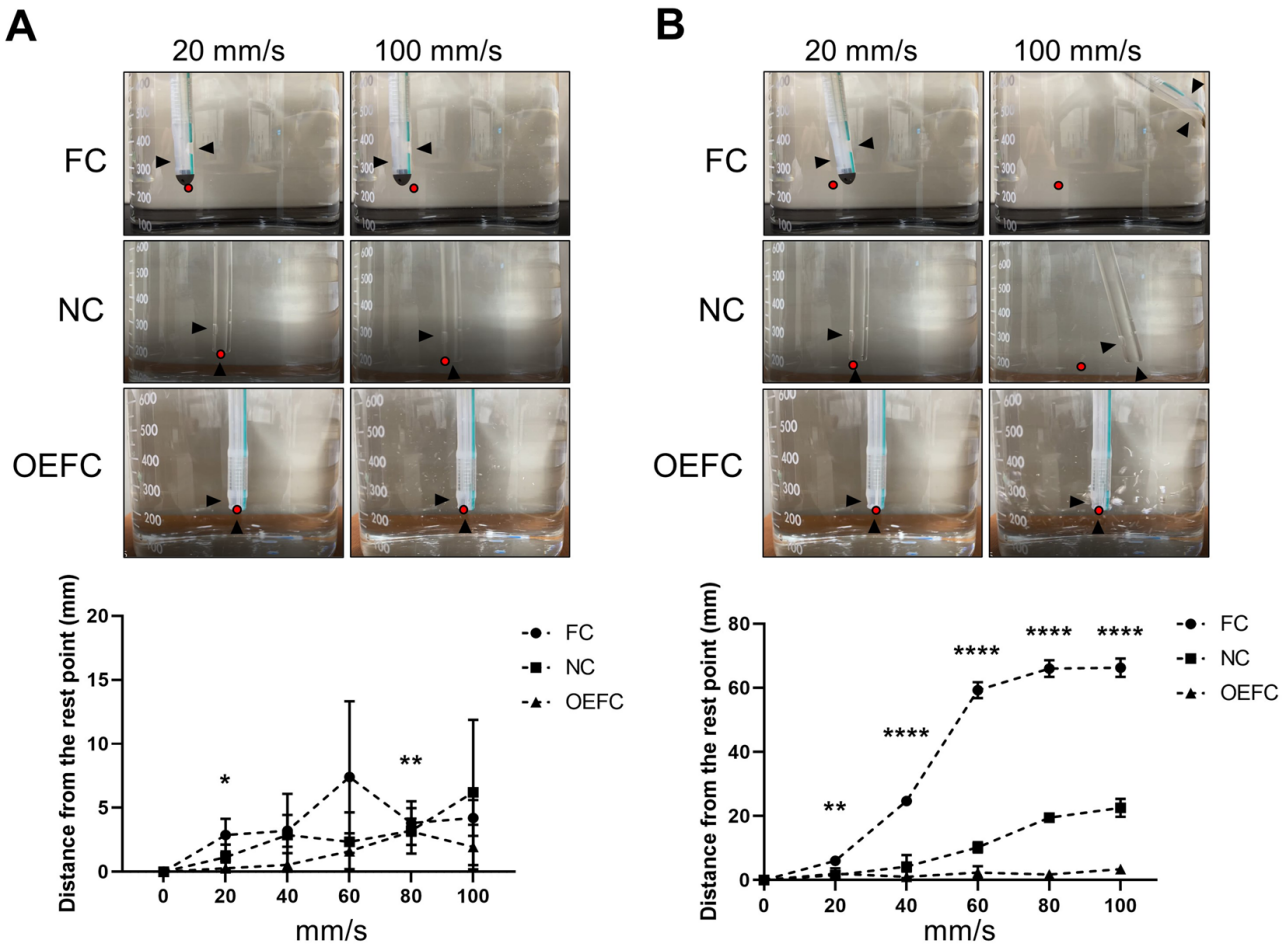
Deviation of each catheter tip. The upper panels of (**A**) show the photographs of each catheter tip when the syringe was pulled at 20 or 100 mm/s. The lower panel of (**A**) shows the distance that catheter tips have moved from their resting points. The upper panels of (**B**) show the photographs of each catheter tip when the syringe was pushed at 20 or 100 mm/s. The lower panel of (**B**) shows the distance that catheter tips have moved from their resting points. All operations were performed with our device at velocities of 20, 40, 60, 80, or 100 mm/s in water. Black arrows indicate the location of each hole and red dots indicate resting points.

Subsequently, we focused on the movement of each catheter tip during one set of predetermined operations with the device at velocities of 20, 40, 60, 80, and 100 mm/s in water. The distances that the catheter tips moved from their resting point are shown (Fig. 4A: pulling term, and Fig. 4B: pushing term). The tips of all the catheters barely moved under the pulling term even on increasing the velocity. Under the pushing term, the tip of FC moved significantly in proportion to its velocity followed by the tip of NC, while the tip of OEFC barely moved even on increasing the velocity (n = 3).

### Efficiency of MBW by using the device

**Figure 5:**
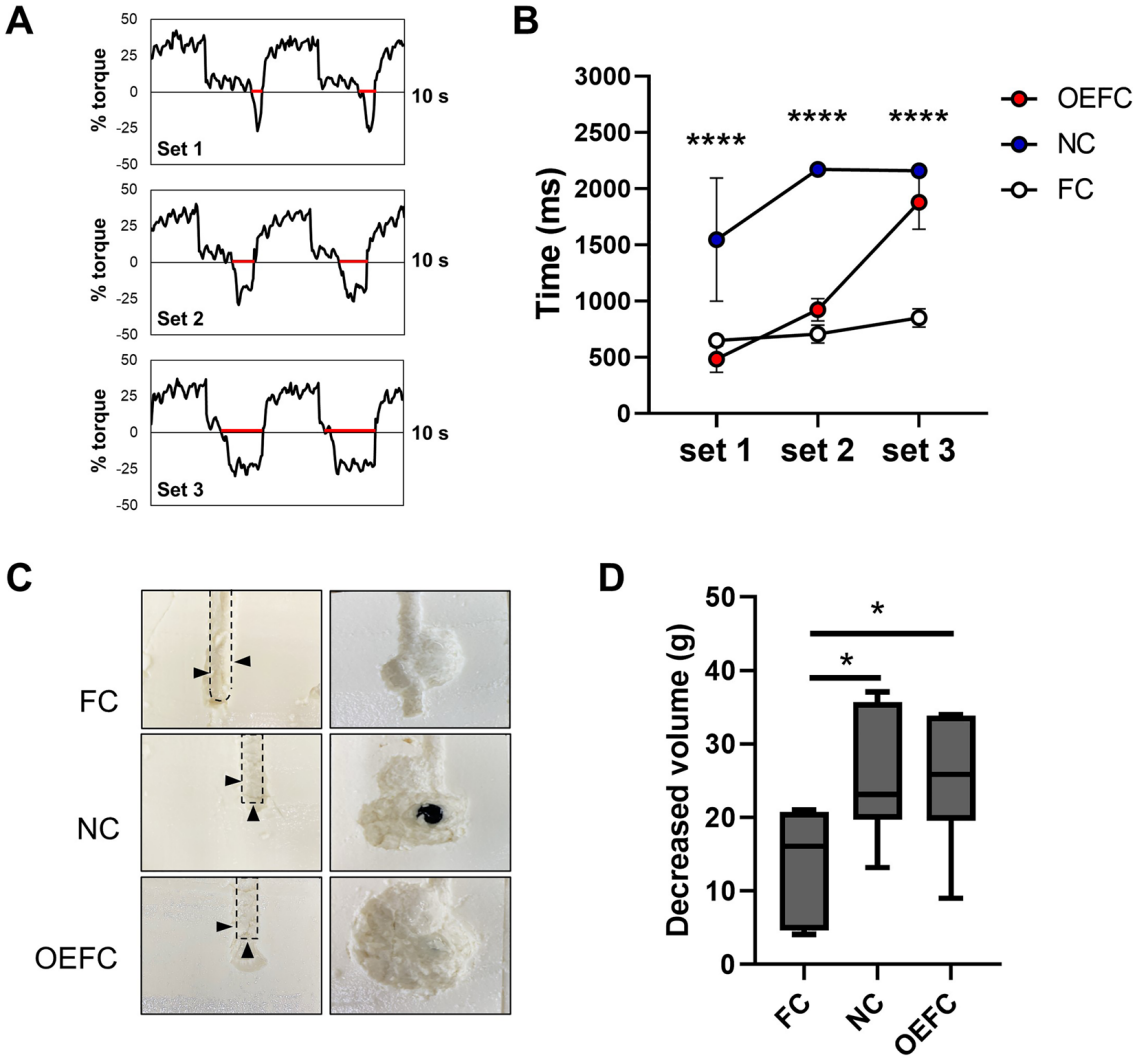
MBW under constant speed. (**A**) Images of the output waveform of the required force (% torque) when the operation was performed using OEFC at 20 mm/s in PBC. Red line shows the time during which the force was required in the minus direction. (**B**) The time to apply the force in the minus direction for each set (n = 6). (**C**) Images of cross sections of PBC before (left side) and after (right side) the three sets of the operations. (**D**) Decreased volume of PBC after the three sets of the operations (n = 6). Black arrows indicate the location of each hole.

To compare the differences in actual clot removal efficiency using catheters attached to the device, a non-penetrating hole was made in the center of the PBC, and the tip of each catheter was inserted into the hole, and the predetermined actions for the device were performed for three consecutive sets (setting the syringe to start operation from the 0 scale, pulling to 30 scale, and pushing to 0 scale, repeating the operation three times was defined as 1 set). Only hard tofu was included in the analysis because soft tofu was too soft and the data was not consistent. The velocity was set to 20 mm/s to eliminate the effect of deviation from the specified position of the tip as much as possible. The output waveform using the OEFC was shown as an example (Fig. 5A). The time to apply the force in the plus direction should have been completely consistent with that in the negative direction, as shown in Fig. 3A. However, negative pressure was expected to be applied in the syringe when it was pulled because the PBC had not collapsed nearly as much in the first set; therefore, the force required in the negative direction was almost zero for most of the time when the syringe was pushed, and then the slight time with the force required in the minus direction passed (shown as a red line in Fig. 5A). In the second and third sets, the time that the force required in the negative direction was expected to have extended as the PBC collapsed enough to irrigate.

The time to apply the force in the minus direction for each set is depicted (Fig. 5B, each n = 6). Focusing on NC, already by the second set, the time that the force required in the minus direction was completely consistent with those for plus direction, as shown in Fig. 3A (between the ranges of 2159 and 2190 ms). However, in FC, the output time in the minus direction did not change much from the first set till the third set.

Image of cross sections of PBC before and after the three sets of the operations is shown (Fig. 5C). NC had a gourd-shaped cross section while OEFC had a nearly circular cross section. NC and OEFC had significantly greater decreased volume of PBC after the three sets of operations than FC (Fig. 5D, each n = 6). Even with OEFC, which had smaller holes than NC, the final decreased volume was the same as that of NC.

### Flow visualization

**Figure 6:**
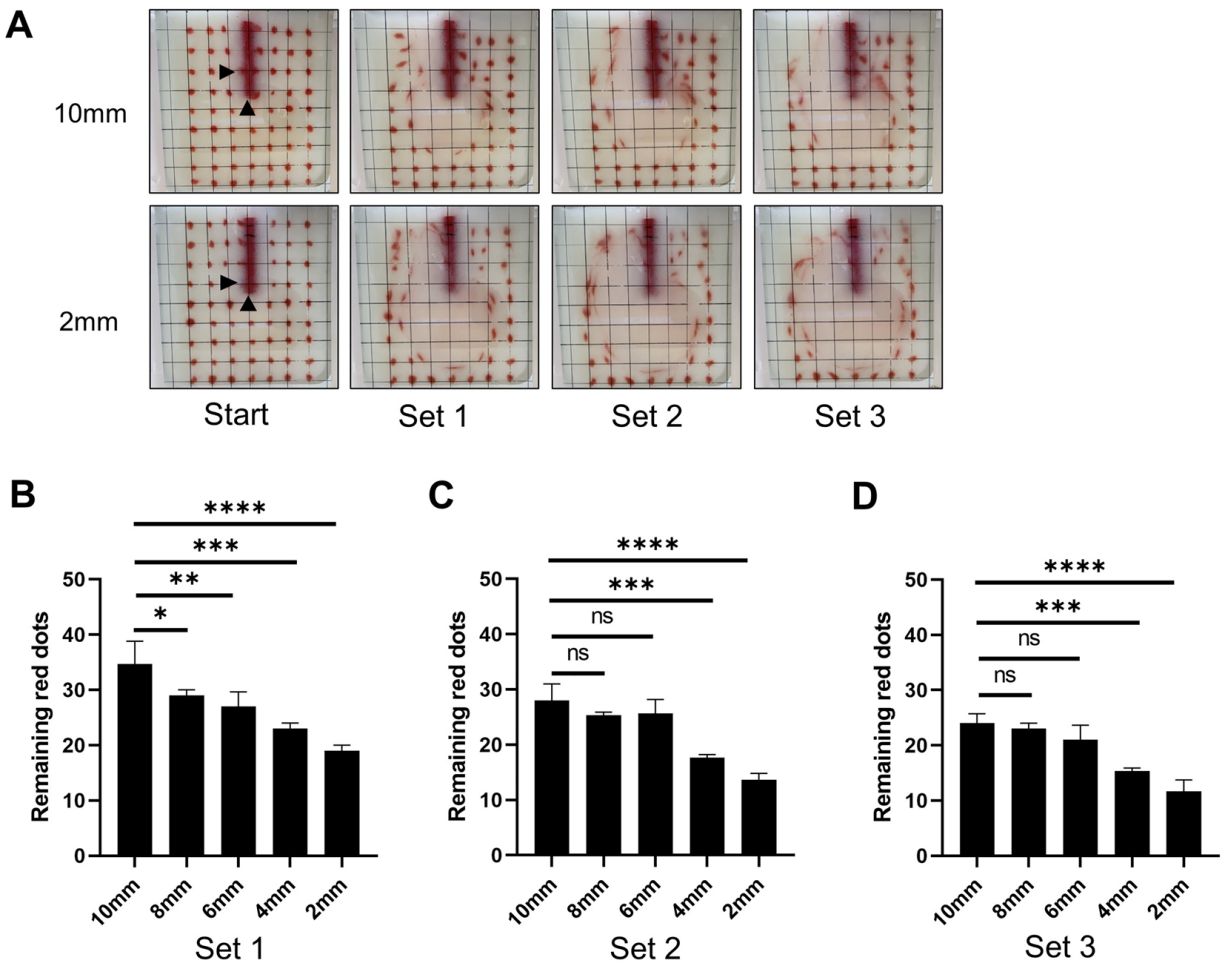
Flow visualization. Modified NC with side holes made at 2, 4, 6, 8, or 10 mm from the tips were used. (**A**) Red dots were drawn on the inside of the front wall of the container to match the intersections of the grid. Images before and after each set using the modified NC with side holes at 2 or 10 mm from the tips under 20 mm/s are shown. Black arrows indicate the location of each hole. (**B**–**D**) The number of red dots remained in the original position were compared between modified NCs after each set.

Based on the results that OEFC with smaller holes had similar efficiency for MBW compared to NC, we hypothesized that the efficiency of MBW would improve when the side hole was closer to the tip. Modified NCs with side holes of 5 mm × 3 mm made at 2, 4, 6, 8, or 10 mm from the tips were fabricated after blocking the original side holes. Fluid motions during the operations using our device and the modified catheters were visualized as shown in the “Materials and methods” section. The velocity was set to 20 mm/s, as described in the previous section. Positions of the red dots before and after the three sets of the operations using the modified catheters with side holes located 2 mm and 10 mm from the tips are shown in Fig. 6A. Out of a total of 63 red dots, the movement of 59 red dots, excluding the four in front of the catheters, as shown on the left side of Fig. 6A was subjected to evaluation. The number of red dots remaining in the original position after the operation with each catheter were compared in each set. The closer the side holes were to the tip, the fewer the number of immobile red dots. Compared to the catheter with side hole made at 10 mm, those at 2 or 4 mm showed significantly reduced remaining red dots in all sets (Fig. 6B–D).

## Discussion

Although MBW is the standard procedure for urinary retention by blood clot formation, the most efficient catheter for MBW has not yet been studied. MBW is sometimes time-consuming, exhausting, and inefficient, which might prolong the patients’ suffering^2^. CATCH-22, a standardized MBW protocol for the management of bladder clot retention, recommended the use of at least 22 Fr catheters, the best infection control catheter manipulation, to assist clearing clot in all quadrants of the bladder, and Clot + 1 L rule^6^. However, there was no mention of recommended catheter types (a balloon Foley catheter with the standard two eyes and rounded tip was used throughout the study to develop the protocol).

We found novel and extremely important findings in this study to compare and evaluate the efficiency of MBW using commercially available urinary catheters and our automatic device for irrigation. On comparing urinary catheters with two holes, an open-ended catheter with a side hole was more efficient than a rounded tip Foley catheter with the standard two eyes.

During MBW, it is difficult to constantly check the position of blood clots in the bladder and the catheter tip by echocardiography. If the tip is misaligned during MBW, it is likely to be difficult to remove blood clots efficiently. We discovered an interesting fact at the first time that the catheter causes a large displacement of the tip position during MBW. An open-ended catheter with a side hole closer to the tip can be used for MBW without deviation from the specified position.

Furthermore, an open-ended catheter with larger holes requires less force for irrigation and can effectively remove blood clots from the early stage of MBW. Finally, if the side hole was closer to the tip, the clot removal efficiency was further improved. Such a catheter, i.e. NC with side hole closer to the tip, can be better to use for MBW if we can use such a catheter in clinical situation. Furthermore, if indwelling in bladder is possible, such as FC or OEFC, continuous bladder irrigation can be performed after MBW^6^, which is even better.

Our device used in this study can perform predetermined movements at a constant speed. A previous report showed that the 60 ml syringe connected with four types of 22 Fr 3-way catheters had an overall velocity from 25 to 29.9 ml/s when 10 operators applied maximum 1-hand pressure through the irrigation and drainage channel of 3-way catheters^9^. Such velocities could be translated to be approximately 40 mm/s in our device.

The alternative method except MBW is to use transurethral instruments under either subdural or general anesthesia such as cystoscope sheath with Ellik evacuator, Toomey syringe^10–12^, or rarely, prostate morcellation device^13^. A method to dissolve and evacuate blood clots using hydrogen peroxide^2^ or alteplase (tissue plasminogen activator)^3^ has also been reported, but such methods cannot be used in all facilities.

Developing a novel catheter based on our findings that can perform MBW in the most efficient manner may reduce procedure time and patient suffering at any institution. However, this study has some limitations. The method for performing MBW by urologists was incomplete in reproducing a stretchable bladder. In a previous report^5^, latex balloons were used as bladder model and mixture of water and Jell-O gelatin powder was used as pseudo clots. We prepared a similar one and tried the experiment, but it did not work. While our method using a 200 ml graduated cylinder and commercially available tofu appears to be highly reproducible, it is desirable to develop a model that more closely resembles an actual bladder and a model that more closely resembles a real blood clot. In addition, the catheters used in this study were only some of those commercially available, and it was only a comparison of catheters with two holes and not with three (or more) holes.

In conclusion, this study provides novel findings by comparing and evaluating the efficiency of MBW using different urinary catheters. The larger the area of the hole in catheter, the less force is required for MBW. Furthermore, the most efficient catheter with two holes needs to be at least open-ended with a side hole closer to the tip for MBW. Further research is needed to determine if the number of holes in the urinary catheter is related to the efficiency of MBW.

## Materials and methods

### Catheters

FC and OEFC were purchased from Fuji systems. NC was purchased from Terumo Corporation. For the purpose of mimicking the difference in hardness of the blood clots, commercially available aoft tofu and hard tofu were used as a PBC because of its ease of availability, uniformity of quality, and moderate hardness. ImageJ software (https://imagej.nih.gov/ij/) was used to measure the area of each hole.

### MBW procedure by urologists

A pseudo clot with a non-penetrating hole punched in the middle was installed in a 200 ml graduated cylinder. Each catheter attached to a 60 ml catheter tip syringe was inserted into the hole, and MBW was performed by urologists in a predetermined manner. The following operation was performed for a total of three consecutive sets: pulling the syringe to 30 ml scale from 0 ml scale and pushing to 0 ml scale, repeating the operation three times, subsequently pulling up to 50 ml scale and discarding the wasted fluids.

The syringe was pulled as far as possible into the position (30 or 50 ml syringe scale) even when there was negative pressure in it. After each set, the waste fluid in the syringe was transferred to another container and the syringe was emptied. the total volume of waste fluid after three sets was defined as wasted fluid’s volume. The weight of the graduated cylinder was measured before and after MBW, and the difference was defined as decreased volume of PBC.

### Device

An automatic irrigation device for a 60 ml catheter tip syringe, which could be used to repeat a predetermined movement at a constant velocity, was fabricated for various quantitative evaluations. The device was purchased from Oriental Motor Co., Ltd. and was composed of the following parts: electric cylinder (EAC4R-E10-AZAKD), motor cables (CC010VZF2), and driver circuit (AZD-KD). Fabrication of the device was commissioned to Monozukuri (Craftwork) Plaza, a university-wide facility at Hiroshima University.

### MBW procedure by device

The moving velocity of the syringe was set at 20, 40, 60, 80, or 100 mm/s. The syringe was set to start operation from the 0 scale, pulled to 30 scale, and pushed to 0 scale, repeating the operation three times. This series of operations was considered in a single set. In this device, the force required to operate the syringe was indicated in % torque, and was measured in ms, up to 10 s (10,000 ms). All operations in water were performed using three different samples for each catheter type. All operations in PBC were performed using six different samples for each catheter type. The weight of the graduated cylinder was measured before and after MBW, and the difference was defined as decreased volume of PBC.

### Visualization of fluid motion

Skim milk at 2.5% concentration with a package of medical thickening agent (Clinico Co., Ltd.) was poured into a transparent rectangular container. The front wall of the container was lined with a grid of lines at 1 cm intervals, and dots were drawn with a red-colored skim milk containing thickening agent on the inside of the front wall to match the intersections of the grid. The catheter was inserted so that it adhered to the inside of the front wall. The predetermined operations by the device were performed, and the movement of the fluid was captured on video. Thereafter, the number of points whose movement from the original position was within 2 mm was counted. These operations were repeated three times and the average value were compared in each set.

### Statistics

Multiple group comparisons were performed using one-way analysis of variance. Unpaired t-tests were used to compare the two groups. All statistical analyses were performed using GraphPad Prism 8 (GraphPad Software, Inc., San Diego, USA). Significant differences are indicated by asterisks as follows: *p < 0.05, **p < 0.01, ***p < 0.001, and ****p < 0.0001.

## Supplementary Information

Supplementary Legends.

Supplementary Video S1.
