# Supplementary material for: Evaluation of urinary catheters for effective manual bladder washout
Source: Sci Rep. 2022 Aug 23;12:14359. doi: 10.1038/s41598-022-18778-5 (PMC9399152; doi:10.1038/s41598-022-18778-5)
Supplement: Supplementary file 1 — Supplementary Legends. [file 41598_2022_18778_MOESM1_ESM.docx]

Supplementary legend

Supplementary video

A scene in which the catheter is moving according to the defined protocol of the movement. Using the software to control the device, we determine the speed of the syringe (20 mm/sec in this case) and the content of the syringe movement (starting from 0, setting the distance to be pulled and pushed).
